# Predicting the Composition of Red Wine Blends Using an Array of Multicomponent Peptide-Based Sensors

**DOI:** 10.3390/molecules20059170

**Published:** 2015-05-20

**Authors:** Eman Ghanem, Helene Hopfer, Andrea Navarro, Maxwell S. Ritzer, Lina Mahmood, Morgan Fredell, Ashley Cubley, Jessica Bolen, Rabia Fattah, Katherine Teasdale, Linh Lieu, Tedmund Chua, Federico Marini, Hildegarde Heymann, Eric V. Anslyn

**Affiliations:** 1 Department of Chemistry, The University of Texas at Austin; 105 E 24th St. Mail Stop A5300, Austin, TX 78712-1224, USA; E-Mail: eman.ghanem@austin.utexas.edu; 2 Department of Viticulture and Enology, University of California; One Shields Ave., Davis, CA 95616-5270, USA; E-Mail: helene.hopfer@gmail.com; 3 Freshman Research Initiative, The University of Texas at Austin, 1 University Station, Mail Stop G2550, Austin, TX 78712, USA; E-Mails: andrea.nav.13@gmail.com (A.N.); mx.ritz@gmail.com (M.S.R.); lina_mahmood@hotmail.com (L.M.); morgan.fredell@gmail.com (M.F.); ashleycubley@gmail.com (A.C.); bolenjb@gmail.com (J.B.); rabia.fattah@gmail.com (R.F.); katherine.teasdale@utexas.edu (K.T.); linh.lieu16@yahoo.com (L.L.); tchua93@gmail.com (T.C.); 4 Department of Chemistry, University of Rome “La Sapienza”, P.le Aldo Moro 5, Rome I-00185, Italy

**Keywords:** differential sensing, supramolecular sensors, wine, blends, tannins

## Abstract

Differential sensing using synthetic receptors as mimics of the mammalian senses of taste and smell is a powerful approach for the analysis of complex mixtures. Herein, we report on the effectiveness of a cross-reactive, supramolecular, peptide-based sensing array in differentiating and predicting the composition of red wine blends. Fifteen blends of Cabernet Sauvignon, Merlot and Cabernet Franc, in addition to the mono varietals, were used in this investigation. Linear Discriminant Analysis (LDA) showed a clear differentiation of blends based on tannin concentration and composition where certain mono varietals like Cabernet Sauvignon seemed to contribute less to the overall characteristics of the blend. Partial Least Squares (PLS) Regression and cross validation were used to build a predictive model for the responses of the receptors to eleven binary blends and the three mono varietals. The optimized model was later used to predict the percentage of each mono varietal in an independent test set composted of four tri-blends with a 15% average error. A partial least square regression model using the mouth-feel and taste descriptive sensory attributes of the wine blends revealed a strong correlation of the receptors to perceived astringency, which is indicative of selective binding to polyphenols in wine.

## 1. Introduction

Wine blending was developed as a European winemaking technique many centuries ago. As the name suggests, blends are made by mixing different mono-varietal wines in certain ratios. Mono-varietal, also called single varietal, wine is made using one grape type. Blending is mainly used to increase the complexity of the wine or enhance its aroma, flavor and texture. For example, Cabernet Sauvignon is considered one of the world’s greatest grapes, but it can be extremely tannic when young. Blending Cabernet Sauvignon with Merlot has been known to attenuate its astringency. Several studies have focused on the sensory and chemical properties of wine blends in comparison to their base wines [1,2,3].

However, wine blending also introduced the possibility of mislabeling and fraud. Inferior or less costly wines could be blended with higher quality wines at unnoticeable ratios and marketed as a more expensive version. This was the case when a French court convicted a wine company, Les Vins Georges Duboeuf, of fraud and attempted fraud for combining grapes in Cru Beaujolais vineyards with grapes from lesser Beaujolais-Villages vineyards [4]. Another case of fraud involved a failed attempt to sell lower-quality local wine as the much more expensive Brunello and Rosso di Montalcino red [5].

Therefore, there is a need to develop techniques to discriminate blends for the purpose of improving quality control and authentication. In previous studies, wines have been analyzed with chemometric techniques, using electronic tongues [6,7,8], electronic noses [9,10,11,12] (to distinguish volatile compounds and which involved differential sensing), Infrared (IR) Spectroscopy [13,14], Nuclear Magnetic Resonance (NMR) [15,16] and Gas Chromatography (GC-MS) [17]. The electronic tongues and noses that were used rely on electrochemical processes involving cross-reactive polymers and various material composites. The use of arrays of cross-reactive receptors with chemometric analysis, in any context, is commonly referred to as differential sensing. Differential sensing is an emerging powerful tool for the analysis of complex mixtures, as it does not require the identification of individual components in the mixture [18].

As a mimic of the natural sense of taste, we have previously developed a class of multi-component, cross reactive supramolecular sensors, and demonstrated their applications in fingerprinting red wines based on their composition of polyphenols [19] and organic acids [20]. Our peptide-based sensing ensembles are composed of a short histidine-containing peptide, a divalent metal ion and a pH colorimetric indicator. Signals from the sensing ensembles are created using the Indicator Displacement Assay (IDA), an analytical assay that was popularized by our group [21]. IDA is based on the displacement of the colorimetric indicator by the analyte, causing a color change that can be measured spectrophotometrically. Not only were the peptidic receptors able to differentiate red wine varietals, they were also shown to discriminate samples of the same wine varietal (Cabernet Sauvignon) that were made using grapes from the same vineyard, but harvested at different times of maturation [22].

In the present investigation, we demonstrate the application of the peptide-based sensing array to differentiate fifteen red wine blends in addition to three base mono varietals (Table 1). The blends are mixtures of Cabernet Sauvignon, Cabernet Franc, and Merlot wines. We also describe how responses of the sensing array to blends made using two mono varietals are used to build a predictive model to quantify the composition of three base wine blends.

## 2. Results and Discussion

### 2.1. Fingerprinting Red Wine Blends

**Scheme 1:**
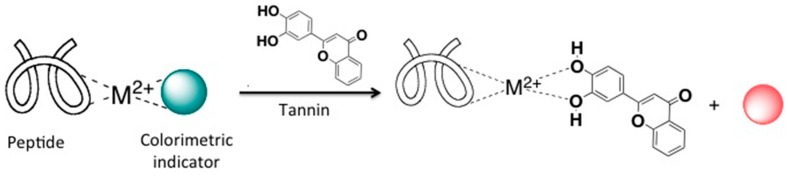
Schematic representation of the Indicator Displacement Assay (IDA) used for the differentiation of tannins in wine.

We have previously demonstrated the application of a multicomponent, peptide-based sensing array to differentiate red wine varietals [19] and Cabernet Sauvignon wines based on harvest time [22]. As mimics of the natural sense of taste, each sensing ensemble is composed of a short peptide, a divalent metal ion and a colorimetric indicator bound via non-covalent interactions. Displacement of the indicator by tannins in wine causes a color change due to the release of the free indicator in solution. This color change is monitored spectrophotometrically (Scheme 1). Although the receptors are assembled via reversible non-covalent interactions, they cannot be regenerated after completion of the assay. There is no drift of sensor components over time. However, all assays are performed in 50% ethanol to ensure solubility of tannins, which may evaporate over time and cause a change of the effective concentration of the sensing ensemble. This is not observed within the current time frame of the assay.

**Figure 1:**
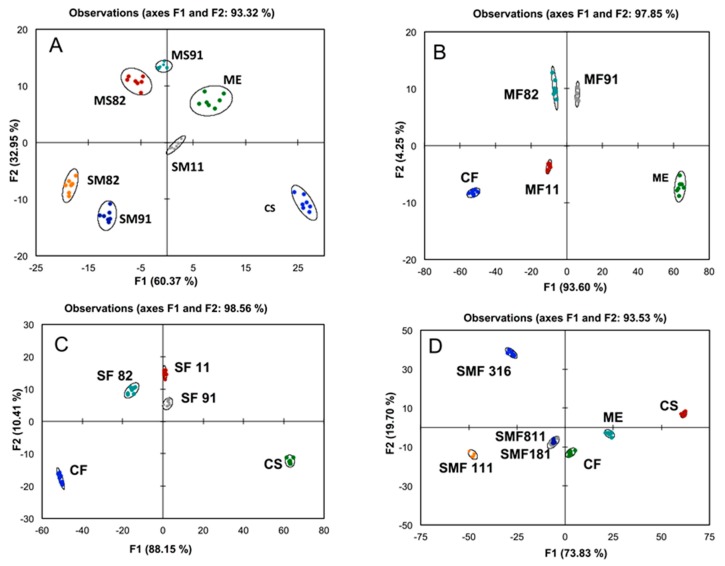
Linear Discriminant Analysis (LDA) score plot of UV-Vis responses of the sensing array to the Cabernet Sauvignon/Merlot blends (**A**), Merlot/Cabernet Franc (**B**), Cabernet Sauvignon/Cabernet Franc (**C**) and tri-blends (**D**). The letters represent the base wines, while the number represent the percentage of each wine. For example, MS28 is a blend of 20% Merlot and 80% Cabernet Sauvignon.

In this paper, we describe a new application for the sensing array to differentiate red wine blends. Nine sensing ensembles were used to measure the responses of three base mono varietals: Cabernet Sauvignon (S), Cabernet Franc (F) and Merlot (M) in addition to fifteen blends composed of different combinations of the base wines. The sensing array is a 3 × 3 combinatorial library of three synthetic short peptides: WAHEDEFF (TT2), WEEHEE (RN8), and FHFPHHF (SEL1) and three combinations of colorimetric indicators and divalent metal ions. The indicator-metal combinations used in this investigation are: Pyrocatechol Violet (PCV)-Cu^2+^, Chrome Azurol s (CAS)-Cu^2+^ and Bromopyrogallo Red (BPR)-Ni^2+^. Responses of the array to the wine blends were measured as the color change caused by displacement of the indicator by the wine tannins. After subtraction of the wine absorbance, the data was analyzed by Linear Discriminant Analysis (LDA) and the resulting canonical variate plots for all seven replicates of each sample are shown in Figure 1. For simplicity, each group of blends is shown with the corresponding base wines. As Figure 1A shows, there is a clear clustering pattern for the Cabernet Sauvignon/Merlot blends with respect to the base wine and the blending ratio. Primarily the first two dimensions explained the variance: F1 and F2, which had values of 60.37% and 32.95%, respectively. This indicates differentiation based on total concentration in addition to composition. In this case, there is a traceable pattern, as blends with Merlot as the predominant wine; MS91 and MS82, were clustered closer to Merlot. This is not the case with Cabernet Sauvignon, which seems to be less influential on the characteristics of the blends. Di-blends containing Cabernet Sauvignon as the major base wine (MS19 and MS28) tend to cluster closer to the other mono varietal, Merlot. Interestingly, this is consistent with the descriptive sensory attributes differentiation patterns for the same blends, where blending Cabernet Sauvignon with other varietals seemed to significantly diminish the perception of its characteristics [1].

A similar trend was observed for the other di-blends made using Cabernet Franc/Merlot (Figure 1B) and Cabernet Franc/Cabernet Sauvignon (Figure 1C). In general, the blends tend to cluster farther away from the mono varietals. This is indicative of a significant change in polyphenol composition, which could be due to oligomerization and cooperative binding of wine tannins to the sensing ensembles [23].

In the case of tri-blends (Figure 1D), Cabernet Franc appeared to contribute more significantly to the properties of blends in comparison to the other two base wines. Blends containing 10% Cabernet Franc and equal amounts of the three base wines (SMF811, SMF181 and SMF111) were clustered closer to Cabernet Franc than Cabernet Sauvignon and Merlot. It is unclear why the blend made of 30% Cabernet Sauvignon, 10% Merlot and 60% Cabernet Franc is distant from the remaining blends and from the three base wines.

### 2.2. Predicting the Composition of Tri-Varietal Red Wine Blends

Partial Least Squares (PLS) Regression was used in this investigation to construct a model for predicting wine composition. PLSR is a statistical method that searches for latent variables (components) that explain as much as possible of the covariance between a set of predictor variables, X, and response variables, Y [24]. The sensor data collected on the binary and ternary mixtures have been arranged in two matrices to be used for the successive step of model building and validation. In particular, the results of the analysis of the three binary mixtures (98 measures, corresponding to 7 replicates of 14 samples coming from Cabernet Sauvignon/Merlot, Cabernet Sauvignon/Cabernet Franc or Merlot/Cabernet Franc blends) have been used as the calibration set, while the 49 measurements of the ternary mixtures (7 replicates for 7 samples) have constituted the test set, to be used for the validation of the predictive model. In order to build a reliable model prior to the chemometric analysis, the 7 measurements for each sample have been averaged and only the mean profiles were used in the successive stages. Since 27 variables have been measured on each blend (the outcomes of the nine sensors at three different wavelength), the predictor matrices X_train_ and X_test_ for the calibration and validation sets had dimensions 14 × 27 and 7 × 27, respectively. The percentages of the three investigated wine varieties in the blends constituted the response matrices Y_train_ and Y_test_, which had dimensions 14 × 3 and 7 × 3, respectively. Indeed, from a modeling standpoint and for the sake of an easier interpretation of the results, the possibility of building a single PLS2 regression model relating the sensor data to the amount of the three investigated wines was preferred over the possibility of modeling each of the responses individually through a PLS1 approach. The difference between the so-called PLS1 and PLS2 approaches lies on the fact that the former considers a single response—meaning, in the present case, that an individual model should have been fitted to predict the amount of each varietal—while the latter accounts for the possibility of modeling altogether all the Y variables.

As anticipated, the training data X_train_ and Y_train_ were used to build the calibration model, whose optimal complexity was selected as the one leading to the lowest Root Mean Square Error (RMSE) in a 7-fold cross-validation procedure. Auto-scaling was used to preprocess the X-block, while the Y-block was only mean centered; eventually, three components were selected for the optimal model, whose characteristic figures of merit are reported in Table 1.

**Table 1:** Model performances in calibration and cross-validation.

	C. Sauvignon		Merlot		C. Franc	
	Calibration	CV ^2^	Calibration	CV ^2^	Calibration	CV ^2^
RMSE ^1^	0.10	0.16	0.065	0.14	0.067	0.15
Bias	0.000	−0.007	0.000	0.003	0.000	−0.004

^1^ RMSE: Root mean square error; ^2^ CV: cross-validation.

**Figure 2:**
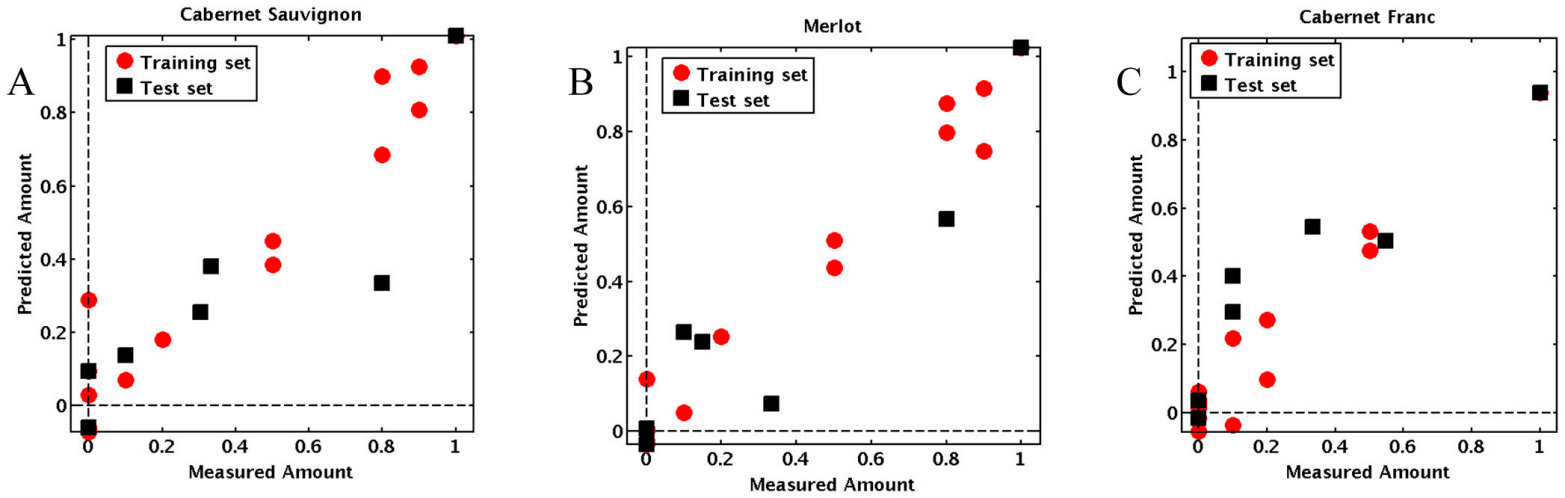
Performance of the Partial Least Squares Regression (PLSR) model using the di-blends as the training set (●) in predicting the percentage of Cabernet Sauvignon (**A**), Merlot (**B**) and Cabernet Franc (**C**) in the tri-blend test set (■). The calculated RMSEP for the model was 0.18, 0.15 and 0.16 for C. Sauvignon, Merlot and C. Franc, respectively.

Successively, the optimal model was used to analyze the validation samples in order to have a more unbiased estimate of its predictive performances than one that could be obtained based on the cross-validation results. Indeed, since cross-validation is used to select the optimal value of model parameters (in this case, the complexity of the PLS model), which are chosen as the ones leading to the lowest error, then estimating the predictive ability only based on this procedure could lead to overoptimistic results. Accordingly, an external test set is often used for this purpose: validation samples are then treated as if they were unknown samples and analyzed using the optimal PLS model. The predictions obtained for the test samples are then compared to the actual values of the responses in order to have a more reliable estimate of the prediction error. The results of the model predictions on the validation samples (tri-blends) are summarized in Table 2 and are graphically represented in Figure 2. As shown in Figure 2, the optimized PLS model allowed predicting the percentage of wine varietals in the blends with very good accuracy.

**Table 2:** Model performances in predicting the percentage of wine varietals in tri-blends.

	C. Sauvignon	Merlot	C. Franc
RMSEP ^1^	0.18	0.15	0.16
Bias	−0.02	−0.01	0.03

^1^ RMSEP: Root mean square error of prediction.

**Figure 3:**
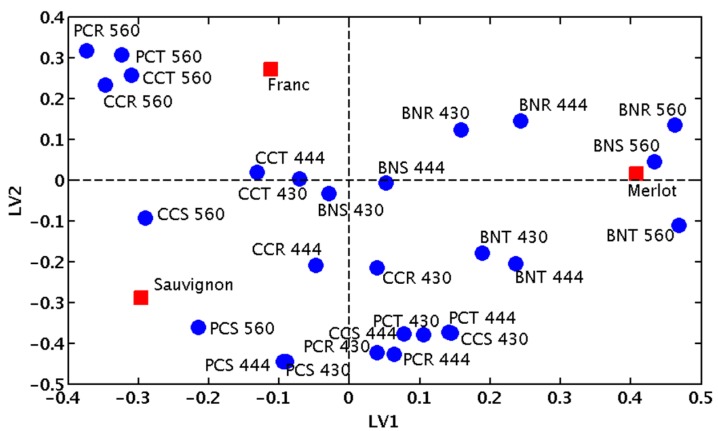
PLSR analysis showing the responses of individual receptors in mono varietals. The first three letters of the sensors represent the first letters of the indictor, metal and peptide, respectively. The number represents the wavelength at which the measurement was taken. For example, BNR 560 represents the ensemble BPR/Ni^2+^/RN8 measured at 560 nm.

Interpretation of the PLS model in terms of the significant variables, *i.e.*, identification of which sensing array responses are most affected by the presence of each wine varietal, can be done by inspecting the loadings biplot reported in Figure 3. For example, the presence of Merlot triggered responses of the sensors BNT 560, BNS 560 and BNR 560, which correspond to the BPR/Ni^2+^ combination bound to the peptides TT2, SEL1 and RN8, respectively, measured at 560 nm. Cabernet Sauvignon produces higher signals in response to the sensors PCS 430 and PCS 444, which correspond to the PCV/Cu^2+/^SEL1 measured at 430 nm and 444 nm, respectively.

### 2.3. Correlation of the Peptide Receptors and Sensory Attributes of Red Wine

**Figure 4:**
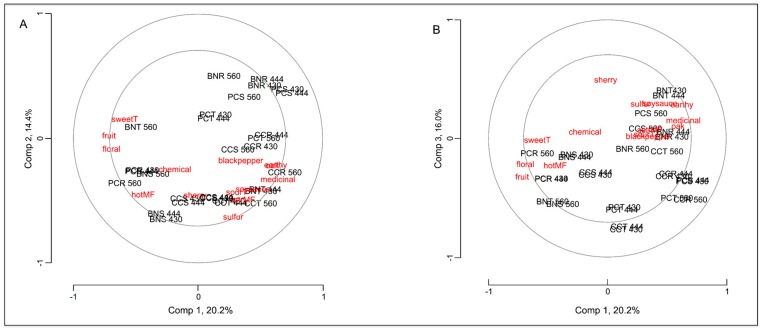
PLSR correlation plot of the predicting variables in black (responses of the peptide assay) and the predicted variables in red (wine sensory attributes) on the first three dimensions; component 1 *vs.* component 2 (**A**) and component 1 *vs.* component 3 (**B**). As shown, the peptide sensors are mostly correlate to taste and mouth feel sensory attributes of red wine and, more specifically, to perceived astringency (astrMF).

To examine the correlation between the peptide-based sensors and the attributes used to describe the sensory effect of wine blending, a PLSR model of the data was performed using results from the peptide assay as *predictors* and the sensory attributes as the *predicted* variables (responses). The correlation between the peptide receptors (in black) and sensory attributes (in red) for the PLS first three dimensions is shown in Figure 4: dimension 1 *vs.* dimension 2 (4A) and dimension 1 *vs.* dimension 3 (4B). As shown in Figure 4, with a few exceptions (like sulfur, floral or fruit), most of the sensory attributes lie close to one or more peptide receptors in the biplot, allowing correlating the former to the instrumental responses. The receptors largely correlate to perceived astringency (astrMF). Figure 4A shows a strong correlation between the receptors CCT 430, CCT 444, CCS 430 and CCS 444 and perceived astringency. This was also true for the receptors BNR 430, BNR 444 and CCS560 (Figure 4B). This is suggestive of selective binding to wine tannins and is consistent with our previous findings [22]. However, it remains unclear and to be investigated whether the receptors discriminate between the different oligomerization states of flavonoids in wine. Grape seed and skin extracts contain a mixture of tannin monomers and small oligos [25], where the degree of polymerization is known to influence perceived astringency [26]. All wine samples used in this investigation were made using grapes harvested at the University of California-Davis owned vineyard. Since temperature is known to influence biosynthesis of polyphenols in grapes [27], future investigations addressing climate and seasonal variation are needed.

## 3. Experimental Section

### 3.1. General

The colorimetric indicators Chrome Azurol S (CAS) (65%), Bromopyrogallol Red (BPR) and Pyrocatechol Violet (PCV) (purity 100%) were purchased from Sigma-Aldrich (St. Louis, MO, USA). Nickel chloride hexahydrate (99.70%), copper (II) sulfate (purity 99.20%) and HEPES buffer were purchased from Fisher Scientific (Pittsburgh, PA, USA). All solid phase peptide synthesis materials were purchased from Novabiochem (San Diego, CA, USA). Wine blends used in this investigation were provided by Heymann’s research group at the University of California-Davis (Davis, CA, USA). The blends were made from the base wines; Cabernet Sauvignon (S), Merlot (M) and Cabernet Franc (F). Composition of the eighteen blends that were tested is shown in Table 3. The letters stand for the respective base wines used to make the blend while the numbers stand for the ratios at which the wines were blended. For example, SM91 is a blend made by mixing 90% Cabernet Sauvignon and 10% Merlot. Total polyphenol concentrations were determined using the Folin-Ciocalteu method. All absorbance measurements were obtained using a Spectra Max Plus 384 plate reader (Molecular Device Inc., Sunnyvale, CA, USA).

**Table 3:** Composition of the Red wine blends used in this investigation.

Number	Label	S	M	F
1	S	100%		
2	M		100%	
3	F			100%
4	SM11	49.6%	50.4%	
5	SM91	90.1%	9.9%	
6	SM82	80.2%	19.8%	
7	SF11	49.4%		50.6%
8	SF91	90.1%		9.9%
9	SF82	80.3%		19.7%
10	MS91	9.9%	90.1%	
11	MS82	19.7%	80.3	
12	MF11		50.7%	49.3%
13	MF91		90.1%	9.9%
14	MF82		80.0%	20.0%
15	SMF111	33.4%	33.2%	33.4%
16	SMF811	80.0%	10.0%	10.0%
17	MSF811	10.1%	79.9%	10.1%
18	Blend	30.4%	14.9%	54.8%

### 3.2. Indicator Displacement Assay of Wine Blends

An array of nine peptide based sensing ensembles was used in this investigation. Each individual receptor was composed of a short, metal binding peptide, a divalent metal ion and a colorimetric indicator. Three peptides were used in our investigation: RN8 (WEEHEE), TT2 (WAHEDEFF), and SEL1 (FHFPHHF). Peptides were synthesized on site as previously described using Prelude solid phase peptide synthesizer, Protein Technologies, Inc. (Tucson, AZ, USA) [19]. The binding ratios of the ensembles have been previously optimized for discrimination of red wine varietals [19]. Table 4 shows the composition of each receptor and the binding ratios used in the assay. All assays were performed in 96-well plates. The final wine concentration was 1% (*v*/*v*) in 50 mM HEPES buffer, pH 7.4. Displacement of the indicators by the tannins in wine was monitored at 430, 400, and 560 nm, the λ_max_ of the free Chrome Azurol S (CAS), Bromopyrogallol Red (BPR) and Pyrocatechol Violet (PCV), respectively. All measurements were taken in seven replicates to ensure reproducibility.

**Table 4:** Composition of individual receptors used in this investigation.

Assembly	Code	Binding Ratio (Indicator:M^2+^:Peptide)	Final Indicator Concentration (mM)
PCV-Cu^2+^-SEL1	PCS	1:1:1	0.075
PCV-Cu^2+^-TT2	PCT	1:1:0.5	0.075
PCV-Cu^2+^-RN8	PCR	1:1:0.5	0.075
CAS-Cu^2+^-SEL1	CCS	1:1:0.5	0.06
CAS-Cu^2+^-TT2	CCT	1:1:0.4	0.06
CAS-Cu^2+^-RN8	CCR	1:1:0.4	0.06
PBR-Ni^2+^-SEL1	PNS	1:1:0.75	0.018
PBR-Ni^2+^-TT2	PNT	1:1:0.4	0.018
PBR-Ni^2+^-RN8	PNR	1:1:1	0.018

### 3.3. Statistical Data Analysis

Linear Discriminant Analysis (LDA) was used for classification of the wine samples. Partial Least Squares Regression (PLSR) was used for the correlation of wine sensory attributes to the peptide-based receptor responses. Data analysis was done using the software XLSTAT (Addinsoft, NY, USA) and R [28]. Absorbance values due to wine without the sensing ensembles were subtracted from the final absorbance change before the multivariate data analysis was performed.

### 3.4. Prediction of the Tri-Blends Composition

Calculations and model building were performed using in-house written routines running under Matlab environment [29]. Averages of seven replicates for fourteen samples: three mono varietals and eleven di-blends were used to construct the predictive model using Partial Least Squares Regression (PLSR). The measured absorbance values were used as predictors (independent variables) while the percentages of the varietals were the responses (dependent variables). Cross validation was used to determine the proper dimensionality (complexity) of the PLSR model. In other words, to determine the number of latent variables (LV) that leads to the lowest RSME. Cross validation is performed by removing a subset of data and building the model using the remaining points. With each iteration, a model is built with varying number of latent variables and an error is calculated for the model. The model was then used to predict the responses for the samples in data subset that was removed, which were not used for building the model. At the end of the cross validation process, all errors are graphed as a function of the number of latent variables and the complexity of the model is determined as the number of LV with the lowest error.

Once optimized, the model was used to test its performance in predicting the responses of a test set. In our case, predicting the composition of tri-blends, which were not used in building the model. The predictions obtained by the model are then compared to the actual (observed) values of the responses to estimate the RMSEP (error of prediction).

## 4. Conclusions

In the present study, we have demonstrated that an array of cross reactive, multicomponent receptors composed of a short peptide, a divalent metal ion, and a colorimetric indicator, can successfully be used to differentiate red wine blends based on tannin composition. This further emphasizes the utility of these sensors in wine analysis, authentication and fraud prevention. Correlation analysis showed that the majority of the sensors correlate well with the taste and mouth-feel descriptive sensory attributes of red wine, and to perceived astringency, in particular. In addition to qualitative tannin analysis, we presented the first quantitative, predictive model for red wine composition. The validated model predicted the percentage of three mono varietals in four independent four tri-blends that were not used to build the model. The quantitative prediction was calculated with a 15% average RMSEP. Our findings set the foundation for developing a multicomponent Electronic Tongue for wine analysis.
